# Proteasomes and Several Aspects of Their Heterogeneity Relevant to Cancer

**DOI:** 10.3389/fonc.2019.00761

**Published:** 2019-08-13

**Authors:** Alexey V. Morozov, Vadim L. Karpov

**Affiliations:** Laboratory of Regulation of Intracellular Proteolysis, W.A. Engelhardt Institute of Molecular Biology RAS, Moscow, Russia

**Keywords:** ubiquitin-proteasome system, constitutive proteasome, immunoproteasome, intermediate proteasome, thymoproteasome, spermatoproteasome, proteasome regulators, cancer

## Abstract

The life of every organism is dependent on the fine-tuned mechanisms of protein synthesis and breakdown. The degradation of most intracellular proteins is performed by the ubiquitin proteasome system (UPS). Proteasomes are central elements of the UPS and represent large multisubunit protein complexes directly responsible for the protein degradation. Accumulating data indicate that there is an intriguing diversity of cellular proteasomes. Different proteasome forms, containing different subunits and attached regulators have been described. In addition, proteasomes specific for a particular tissue were identified. Cancer cells are highly dependent on the proper functioning of the UPS in general, and proteasomes in particular. At the same time, the information regarding the role of different proteasome forms in cancer is limited. This review describes the functional and structural heterogeneity of proteasomes, their association with cancer as well as several established and novel proteasome-directed therapeutic strategies.

## Introduction

The proteasomes degrade the majority of intracellular proteins and represent a crucial element of the ubiquitin-proteasome system (UPS), which is involved in almost all cellular metabolic processes (1, 2). Flexibility of the UPS is necessary for cells to survive and adapt to various conditions. Therefore, the system has different levels of organization (3). Focusing on proteasomes these can include the following: (1) structural and catalytic subunit diversity (4), (2) presence or absence of different regulators (5), (3) post-translational modifications of proteasome subunits (6, 7), and (4) interactions with different protein cofactors (other than regulators) (2, 8, 9). The first three levels create different forms or subtypes of proteasomes. Various forms of proteasomes can be found in a single cell, their ratios are changing from tissue to tissue and influenced by various stimuli (5, 10). Moreover, roles played by one proteasome form may be not or incompletely covered by other forms. Recent findings made a breakthrough in the quantitative analysis of proteasome form diversity in various cells, including cancer cells (5, 6, 11–16). The fundamental role of proteasomes in the maintenance of homeostasis has made them attractive targets for anti-cancer therapy, and proteasome inhibitors are used to treat multiple myeloma and mantle cell lymphoma (17, 18). However, their efficacy is limited especially against solid tumors. Emerging data highlight the increasing role of different proteasome forms in cancer, which creates novel opportunities for therapeutic intervention at different proteasome organization levels. The review describes the diversity of proteasomes and certain aspects of proteasome heterogeneity relevant to cancer.

## 20S Proteasome

**Figure 1:**
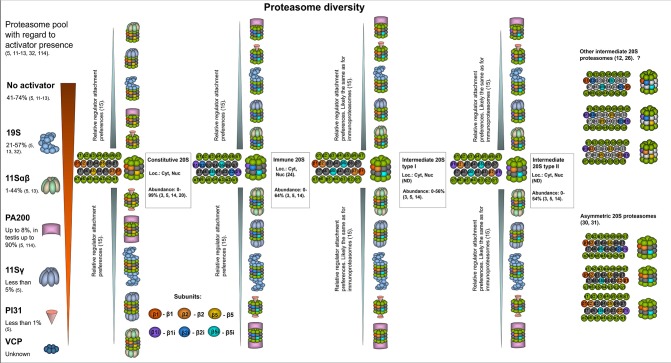
The structural diversity of proteasomes. There are several levels of proteasome organization (3). Proteasomes differ by composition of subunits forming 20S core particles. Major 20S proteasomes include: constitutive proteasomes, intermediate proteasomes of type I and II, immunoproteasomes. The constitutive 20S proteasomes contain constitutive catalytic subunits: β1, β2, and β5. These proteasomes are found both in the nucleus and the cytoplasm. Up to 85% of all cellular proteasomes contain constitutive 20S cores (3, 5, 14, 20, 21). Thus, constitutive 20S proteasome appears to be the most abundant form of 20S core particle found in various tissues including heart, kidney, skeletal muscles, brain as well as in different cell lines of embryonal or cancer origin (14, 20, 21). 20S immunoproteasome contain immune subunits β1i (LMP2), β2i (MECL-1), and β5i (LMP7) instead of constitutive β1, β2, β5, correspondingly. These complexes were also found both in the nucleus and the cytoplasm (22). Normally, immunoproteasomes are predominantly detected (up to 64% of total proteasome pool) in cells of the immune system as well as in the small bowel and colon, however can be upregulated in many other cells following exposure to the immune cytokines or in stress conditions (3, 14, 23, 24). The intermediate proteasomes contain the immune catalytic subunits together with the constitutive ones. The subcellular localization of intermediate proteasomes was not carefully addressed, and these complexes may be present both in the nucleus and the cytoplasm. The type I (β5i) intermediate proteasomes contain β1, β2, and β5i subunits. In the cellular proteasome pool type I intermediate proteasomes constitute from almost none to more than 50% depending on the tissue and cell type (5, 14). These proteasomes are found in large numbers in the liver, small intestine, colon, muscles (12, 14, 25), dendritic cells (14), as well as in the cells of acute promyelocytic leukemia NB4 and histiocytic lymphoma U937 cell lines (5). The type II intermediate proteasomes contain β1i/β2/β5i catalytic subunits and are abundantly found in monocytes representing up to 54% of the proteasome pool (14). In addition, these proteasomes were detected in several cancer cell lines including acute myelogenous leukemia line KG1a (3, 5). Experimental data indicate that other forms of intermediate proteasomes can be formed e.g., complexes with β1/β2i/β5i (12), β1i/β2i/β5 (26), β1i/β2/β5 (26). Some of them were detected in unnatural conditions (in β5i^−/−^ mice, in cells with β5 overexpression etc.), thus their presence *in vivo* is debated. Moreover, some of these forms are against rules of cooperative assembly (27–29). Nevertheless, their presence in certain situations cannot be entirely excluded. Another level of 20S complex diversity can arise from the fact that each 20S proteasome contain two copies of every subunit imbedded in different rings thus, theoretically, asymmetric proteasomes containing one immune and one constitutive beta-subunit in the same complex can be formed (30, 31). Still this could be a temperate occasional event in the process of proteasome pool reorganization following the stimulation with cytokines. Another level of cellular proteasome organization is a presence of an activator. By several authors it was demonstrated that the majority (depending on the tissue or cell type from 41 to 74%) of cellular proteasomes are “free” and do not bare an activator (5, 13). The activator that is most frequently attached to the 20S proteasomes is the 19S complex. It was found bound to from ~21–57% of 20S complexes in various cells and tissues (5, 13, 32). Proteasomes with 19S activator were detected in the cytoplasm and the nucleus. The 11Sαβ is a second most frequent cytoplasmic proteasome activator and from a single per cent to 44% of cellular proteasomes may be capped with it (5, 13). PA200 and 11Sγ are the regulators, that associate with proteasomes preferentially in the nucleus covering generally up to 8% and <5% of total proteasome pool, respectively (5). PI31 proteasome inhibitor associates with ~1% of cellular 20S proteasomes (5). Whether VCP associates with 20S core particle in mammalian cells and how frequently, is not entirely clear. Interestingly, preferential association of proteasome activators with different 20S core proteasomes was recently shown (15). It was demonstrated that 11Sαβ and 11Sγ “prefer” immune and likely intermediate 20S proteasomes, while PA200 and PI31—the constitutive ones, 19S binds all the complexes equally (15). At the same time, several reports argue preferential association of certain activators with constitutive or immune 20S proteasome (33). The heterogeneity of cellular proteasomes is even greater and the image does not include various post-translational modifications of proteasomes (7) as well as proteasome interacting proteins with regulatory functions (3). Question mark indicates that the presence of a particular proteasome form is uncertain.

The 20S proteasome, or 20S core particle (20S CP), is a 150 Å to 115 Å, 700 kDa complex, assembled from 28 proteins arranged in four heptameric rings (19). The rings lay one on another and are composed of either seven alpha- or seven beta-subunits. Two alpha rings are external, while two beta rings reside between them to form α_1−7_β_1−7_β_1−7_α_1−7_ arrangement (Figure 1) (19, 34). The N-terminal portions of the alpha-subunits form so-called gates that close the central pore and oppose occasional penetration of substrates into the internal chambers of the proteasome. Inside the proteasome, protein hydrolysis is executed by the N-terminal threonines of three of the seven beta-subunits, which mediate nucleophilic attack of the substrate peptide bonds (35). The three catalytically active beta-subunits have identical peptide bond hydrolyzing mechanisms; however, they demonstrate different sequence specificity; thus, proteasomes have three major proteolytic activities, and a particular subunit is responsible for each particular activity.

It was assumed that the closed conformation of the alpha-subunit gates causes the majority of 20S proteasomes to be latent and incapable of degrading proteins without being activated by activators (described below). However, it was demonstrated that 25% of yeast 20S proteasomes have open gates (36, 37). Moreover, engagement of the proteasome catalytic site in substrate degradation induces allosteric gate opening and reverses the ratio of open-to-closed 20S complexes (36, 37). Furthermore, proteins with so-called intrinsically disordered regions can be degraded by 20S proteasomes (9, 38–40). The 20S proteasomes participate in stress adaptation by degrading oxidized and damaged proteins (41–43). In addition, 20S proteasomes are involved in interneuronal communication (44). They can also digest a certain part of the protein leaving stable products with important biological functions (39, 45–48).

“Free” 20S complexes (proteasomes with no attached activators) is the most abundant form of proteasome, comprising 47–74% of all cellular proteasomes (Figure 1) (5, 11–13); interestingly, very low numbers of these complexes have been reported in rodent brains (16). At the same time, all cellular proteasomes (both free and bound to activators) contain the 20S core. However, the cores differ by their subunit composition.

### Constitutive Proteasomes

The catalytic beta-subunits with rare exclusions determine the forms otherwise subtypes of 20S proteasomes. The constitutive (sometimes also referred as standard) 20S proteasome (cPs) contains the β1(Y) (*PSMB6*), β2 (Z) (*PSMB7*), and β5 (X) (*PSMB5*) catalytic subunits, which mediate cleavage after acidic (β1), basic (β2), or hydrophobic (β5) amino acids, thus demonstrating caspase-like, trypsin-like and chymotrypsin-like activities, respectively (49–51). Constitutive proteasomes are the most frequent form of 20S core found in many different tissues, including heart, brain, liver, kidney, skeletal muscles, and in different cell lines, including HeLa, HEK293, HCT116, EB81-MEL, NCI-H460, MRC-5, LB1751-MEL, NB4, SK23-MEL, U2OS, L363, and RKO (3, 5, 14, 20, 21). Specifically, up to 85% of all cellular proteasomes (free or capped with different activators) contain constitutive 20S cores (Figure 1) (3, 5, 14, 20, 21). Moreover, only cPs were found in erythrocytes, Huh7, and HeLa cells (30, 52, 53).

Constitutive proteasomes are upregulated in different cancer types, reflecting an increased demand for the degradation of proteins and the maintenance of metabolism. This creates vulnerability within the tumor, and proteasome inhibitor-based therapy was developed as a result. Proteasome inhibitors, however, demonstrate different efficacies depending on tumor type, and drug resistance is frequently observed (54). Resistance mechanisms include the activation of autophagy and pro-survival pathways, the alteration of proteasome expression and the introduction of mutations in beta-subunit genes (54, 55). Interestingly, in a recent paper, mutations in genes encoding catalytic beta subunits of proteasomes, were detected in proteasome inhibitor-treated patient with multiply myeloma, highlighting the role of mutations in development of the drug resistance (56). Moreover, the inhibition of β2 and β5 was shown to promote epithelial-mesenchymal transition (EMT) in immortalized human mammary epithelial cells, which acquired self-renewal capability and cancer stem cell features (57). Furthermore, low expression of *PSMB5* was associated with poor prognosis in breast cancer patients. These data indicate that proteasome inhibitors may induce EMT in certain tumors and facilitate their growth (57).

Proteasomes degrade proteins and generate peptides that are consequently exposed on the cellular surface and recognized by T cells. Constitutive proteasomes more efficiently than other proteasomes produce immunogenic peptides from several differentiation antigens (58). These include the melanoma antigen gp100 peptides: gp100_209−217_, spliced peptides gp100_40−42and47−52_, gp100_195−202and191or192_ (59, 60); the tyrosinase peptides: tyrosinase_369−377_ and tyrosinase_368−373and336−340_ (59, 60); and the MART-1_26−35_ peptide (60, 61). Hence, the balance between proteasome forms in tumor cells should be considered when therapy directed against differentiation antigens is developed. Indeed, immunotherapy of melanoma patients with mature dendritic cells that were artificially enriched with cPs and transfected with RNAs encoding the cancer antigens MART-1, MAGE-3, gp100, and tyrosinase was more efficient than therapy with transfected wild-type dendritic cells, which mostly expressed immunoproteasomes (62).

### Immunoproteasomes

The immunoproteasomes (iPs) contain the so-called immune catalytic subunits: β1i (LMP2), β2i (MECL-1), and β5i (LMP7) in place of β1, β2, and β5, correspondingly (63–69). The β2i is encoded by the *PSMB10* gene. The β1i- and β5i- encoding genes *PSMB9* and *PSMB8* are localized within the MHC class II region, which is the one reason for the term “immuno” (64–66, 70). Another reason is that the synthesis of these subunits is driven by IFN-γ via the Jak/Stat pathway (66, 70–73). Nevertheless, other molecules can stimulate production of immune subunits, including TNF-α (66), LPS (74), type I interferons (31, 75), nitric oxide (76), and glycoxidized proteins (77). Due to structural differences in the substrate binding pockets of the immune subunits, iPs have decreased caspase- and increased chymotrypsin- and trypsin-like activities (19, 78, 79). Thus, compared with cPs, they have altered cleavage preferences and a spectrum of generated peptides. Interestingly, Mishto et al. demonstrated that constitutive and immunoproteasomes demonstrate quantitative rather than qualitative differences in the spectrum of produced peptides (80). However, in a more recent study it was demonstrated that although both proteasome forms share most cleavage sites, 32% of the cleavage sites of immunoproteasomes and 19.5% of the cleavage sites of constitutive proteasomes do not overlap (81). Immunoproteasomes effectively generate peptides with hydrophobic C-termini compatible with major histocompatibility complex class I (MHC I); hence, they play important roles in inflammation and immune reactions (4, 82–84). IPs also participate in stress adaptation and degradation of damaged proteins (4, 41, 42, 85). Moreover, these proteasomes are involved in the regulation of signaling pathways, in the control of T lymphocytes expansion (86), visual transmission (87), in the maintenance of pluripotency of stem cells (88), muscle differentiation (89), and the production of cytokines (90, 91). Furthermore, iPs influence the transcription of more than 8,000 different genes in dendritic cells (92). Compared to constitutive proteasomes, iPs have a shorter half-life (27 vs. 133 h), which is understandable since in the majority of tissues they are upregulated in response to stress, inflammation, infection and subsequent cytokine exposure but are no longer needed in large quantities when the stress is ceased (93). Conversely, in immune cells, cells of the small bowel and colon mucosa cells, iPs normally comprise up to two-thirds of the cellular proteasomes (14, 22–24). Moreover, almost all proteasomes in T-lymphocytes are iPs (94) and high numbers of immunoproteasomes were detected in KG1a and THP-1 cell lines (5). The involvement of iPs in the pathogenesis of autoimmune diseases, neurodegenerative diseases and cancer has been described (24, 91, 95–97); thus, immunoproteasome-specific inhibitors have been developed and tested as anti-cancer drugs (98).

The overexpression of immunoproteasome genes is frequently observed in different cancer types and indicates a better prognosis for breast cancer patients (99, 100). This may be attributed to the infiltration of tumors with CD8+ lymphocytes (99, 100). These cells contain iPs but also, via the secretion of IFN-γ, can induce iP expression in cancer cells and thereby modulate the spectrum of presented peptides and hence their immune “visibility” (58). Concordantly low β5i levels have been associated with reduced disease-free survival in non-small cell lung carcinoma (101). However, an analysis of patient tumor samples showed that β5i levels do not always correlate with lymphatic infiltration, indicating other reasons for immunoproteasome subunit overexpression in certain tumors. Indeed, the interplay between the STAT1 and STAT3 transcription factors as well as enhancer hypermethylation seems to influence the regulation of iP subunit expression in non-small lung carcinoma cells (101). This became evident after a comparative analysis of carcinoma cell lines with epithelial-like and mesenchymal-like morphology. The latter showed a significantly decreased level of iP subunit expression and a decline in the amount and diversity of MHC I-presented peptides. This can facilitate tumor escape from recognition by CD8+ T cells. Moreover, TGF-β-induced EMT promoted iP content decrease (101). These results are congruent with data demonstrating that the inhibition of constitutive proteasome subunits induces EMT and stimulates the acquisition of cancer stem cell characteristics (57). Therefore, immunoproteasome-specific inhibitors likely may also induce EMT and the generation of specific cancer cell populations with stem cell properties. However, treatment with one inhibitor can make cancer cells sensitive to lower concentrations of another inhibitor. For example, β2/β2i subunit inhibitor sensitized myeloma cells to the β5i subunit inhibitor ONX-914 (102). Thus, targeting different forms of proteasomes present in cells limits cellular capabilities to compensate decreased levels of proteolysis and allows reduction of applied drug concentrations. Interestingly, cancer cells with high numbers of iPs were found to be more sensitive to the proteasome inhibitors bortezomib and MG132 which inhibit both constitutive and immunoproteasomes (99).

Conversely, elevated iP expression can “help” tumors. Immunoproteasomes facilitate adaptation to stressors (41, 85). Accordingly, demethylation-dependent immunoproteasome overexpression in myeloid leukemia cells was associated with increased cellular resistance and survival in conditions of oxidative stress (99). Another example is the generation of the immunodominant MART-1_26−35_ epitope in tumor cells. It was shown that it is impaired in melanoma and HeLa cells following the upregulation of immunoproteasome subunits (especially β2i and β1i). This reduces the efficacy of epitope presentation and can facilitate tumor escape from immune pressure (61, 103). Considering the role of immunoproteasomes in inflammation, the modulation of iP activity can impact inflammation-induced cancer development. Indeed, the inhibition of β5i leads to a reduction in tumor numbers in a mouse model of colitis-associated cancer (91), while the inhibition of β1i attenuated tumor growth in a mouse model of prostate cancer (104).

In fact, immunoproteasome-mediated effects could instead be associated with the presence of intermediate proteasomes, since these forms of proteasomes are not always separated.

### Intermediate Proteasomes

Incorporation of immune subunits into the assembling 20S proteasome is considered to proceed according to the rules of cooperative assembly, involving coincorporation of β1i and β2i following preferential inclusion of β5i (27–29). However, from one-third to one-half of the proteasomes in liver, colon, small intestine and kidneys contain both constitutive and immune subunits being intermediate between cPs and iPs (12, 14, 25, 29, 30, 105, 106). There are two dominant types of intermediate 20S proteasomes (intPs).

*Type I* (β5i) intPs have β1/β2/β5i architecture. These proteasomes have slightly different activity than constitutive proteasomes or iPs (12) and demonstrate increased chymotrypsin- and trypsin-like activity, comparing to cPs (14, 107, 108). The ratio of type I intermediate proteasomes to other 20S proteasome forms could be as low as 1–2% or could be 50% or higher depending on the tissue type (5, 14). β5i intermediate proteasomes were reported in the liver, kidneys, small intestine, colon, dendritic cells, and U937 cells [5, 14, 102].

*Type II* (β1i and β5i) intPs contain β1i/β2/β5i catalytic subunits (109). Concordantly with subunit set, these proteasomes have increased chymotrypsin-like activity and trypsin-like activity but low caspase-like activity in comparison with cPs (14). Type II intermediate proteasomes constitute more than half of the proteasome pool in monocytes (14) and are found in large numbers in the cell line KG1a (5).

#### Additional Types of intPs

It is generally assumed that β5 is preferentially incorporated into proteasome precursors containing β1/β2 (29, 30, 105). However, Joeris and coauthors demonstrated that the 20S core particle with architecture β1i/β2i/β5 can be found in embryonic fibroblasts under IFN-γ stimulation and in conditions of overexpressed β5. In addition, authors revealed proteasomes with catalytic subunit set β1i/β2/β5 in livers and β1i/β2i/β5 in spleens of healthy β5i^−/−^ mice (26). Moreover, β1i/β2i/β5 containing proteasomes were detected in livers upon infection of animals with the *Listeria monocytogenes* (26). It should be mentioned that complexes with β1i/β2i/β5 architecture were also previously found by another group in thymus and spleen of the β5i^−/−^ mice (110). On the contrary proteasomes containing β2i/β5 were not detected in nine cell lines by Fabre et al. (15). Dahlmann and coauthors by means of chromatographic separation of rat muscle and spleen tissue lysates discovered additional forms of intermediate proteasomes with β1/β2i/β5i as well as (β1 and β1i)/β2/β5i and (β1 and β1i)/β2i/β5i architecture (12).

Here it should be emphasized that each 20S proteasome contain two beta subunit rings, thus all subunits are presented in duplicates. This leaves a possibility that one beta ring would contain the constitutive beta subunit, while the other—the immune subunit. In fact, the existence of such 20S proteasomes was reported in several publications. For example, following cytokine stimulation 20S proteasomes containing both β5i and β5 were revealed by Freudenburg and coauthors (31), while complexes bearing β1i and β1 simultaneously were demonstrated by Klare et al. (30). These kinds of intermediate proteasomes were denoted as asymmetrical 20S proteasomes (Figure 1). Furthermore, it was proposed that 10 different subtypes of assymetrical intermediate 20S proteasomes might exist (30). The presence of some subtypes was never shown and is doubtful, however one cannot exclude that such complexes still can be assembled though in a very limited number of cases (28, 29, 109).

IntPs broaden the repertoire of generated peptides. Interestingly, they produce unique peptides when processing certain tumor antigens, including proteins belonging to a subclass of cancer testis antigens that are promising anti-cancer therapy targets (14, 111). Indeed, it has been revealed that certain peptides from proteins of the MAGE (Melanoma Antigen Gene) family are predominantly generated by β1i-β5i intermediate proteasomes including MAGE-A10_254−262_ and MAGE-C2_191−200_ (14, 60), while MAGE-A3_271−279_ peptides are produced mostly by β5i intPs (14, 60, 112). Nevertheless, several antigenic peptides, such as MAGE-A3_114−122_ and MAGE-C2_42−50_, are generated by intPs and iPs with comparable efficiency (60). Production of such peptides leads to the recognition of cells by cytotoxic lymphocytes and thus strongly augments anti-tumor immunity. The expression of intPs in tumors was insufficiently studied, although it may be partially regulated by factors which influence immunoproteasome abundance. IntPs have been detected in the lung carcinoma NCI-H460, myeloma L363, colorectal carcinoma HCT 116, colon carcinoma RKO, cervical cancer HeLa, histiocytic lymphoma U937, acute myelogenous leukemia KG-1a, acute promyelocytic leukemia NB4, and osteosarcoma U-2 OS cell lines and in several melanoma cell lines, including UKRV-Mel-21a, Ma-Mel-63a, A375, EB81-MEL, LB-39-MEL, LB1751-MEL, low level SK23-MEL, UKRV-Mel-6a, UKRV-Mel-15a, and Ma-Mel-86a (5, 14, 61).

### Thymoproteasomes

**Figure 2:**
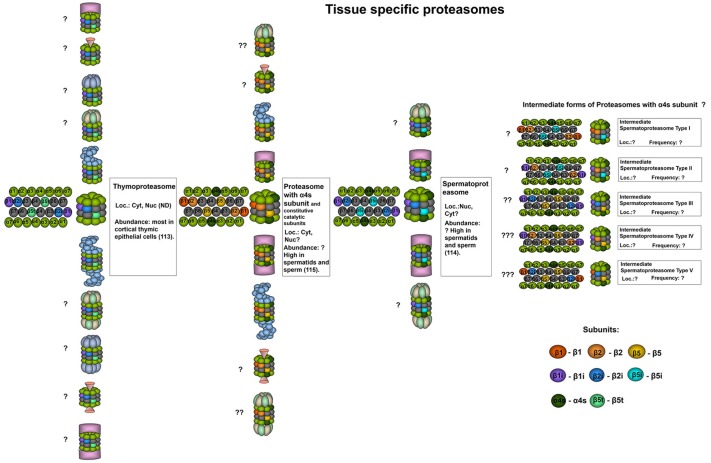
Tissue specific proteasomes. Thymoproteasomes contain immune subunits β1i and β2i and a specific catalytic subunit β5t (113). These proteasomes are found exclusively in the thymus and can represent up to 20% of total proteasome pool there, concentrating specifically in cortical thymic epithelial cells where tP is the dominate form of the proteasome (113). Another unique 20S proteasome form is found in the testis. These proteasomes contain α4s subunit together with constitutive, immune or, probably, catalytic β-subunits of both types. Proteasomes with α4s and immune catalytic subunits were named spermatoproteasomes (114). The proportion of proteasomes containing α4s to proteasomes with constitutive α4 subunit in sperm could be as high as 80% (115). Question mark indicates that the presence of a particular proteasome form is uncertain.

The unique form of 20S proteasomes known as thymoproteasomes (tPs) is present in cortical thymic epithelial cells (Figure 2) (113). TPs contain immune subunits β1i and β2i, and the distinctive catalytic subunit β5t (*PSMB11*) (113). Together with other factors, *PSMB11* gene expression is regulated by the transcription factor Foxn1 (116). Comparing to cPs and iPs thymoproteasomes exhibit significantly [60–70%] decreased chymotrypsin-like activity due to the presence of hydrophilic amino acids in the substrate binding pocket of the β5t (113). This peculiarity of tP enables it to produce specific peptides (117–119) with optimal affinity for T cell receptors (TCR) to effectively promote positive selection of lymphocytes (119). Indeed, β5t^−/−^ mice have decreased levels of CD8+ single positive lymphocytes (113). Furthermore, CD8+ T cells generated in thymus that lacks tPs demonstrated diminished TCR responsiveness, decreased numbers of naïve peripheral cells and altered responses to infection (120). Hence, thymoproteasomes affect not only the fate of T cells during positive selection but also determine the functionality of mature lymphocytes (120).

The expression of β5t has been reported only in thymomas (121). Immunohistochemical analysis of thymoma (subtypes A, AB, B1, B2, and B3) and thymic carcinoma tissues revealed β5t expression in 35% of AB subtype thymoma and 95% of B subtype thymoma but not in A subtype thymoma or thymic carcinoma samples (121, 122). Thus, β5t could be utilized as a marker for thymomas and could be used to discriminate between B3 subtype thymoma and thymic carcinoma (121, 123). Moreover, β5t expression was shown in cervical ectopic thymomas and in 10 out of 10 cases in which remnants of thymic tissue were found in the neck, indicating that β5t expression can serve as a marker of rare ectopic thymomas at different sites in the body, including the chest wall, pleura, lung, and heart (124). Finally, the authors proposed that aberrant expression of β5t in thymomas may induce autoimmune diseases through the generation of a self-reacting population of lymphocytes (123). Indeed, it would be interesting to compare the peptide patterns displayed on the surfaces of cells with normal and dysregulated β5t expression.

### Spermatoproteasomes

Spermatoproteasomes (sPs) (Figure 2) are testis-specific and are characterized by the presence of α4s subunit (*PSMA8*), they have a temporal expression profile and have been described solely in spermatocytes, spermatids and sperm (24, 114, 115, 125). The incorporation of α4s and α4 into the 20S proteasome is mutually exclusive and does not alter the catalytic activity of the complex (115). Qian et al. reported immune catalytic beta subunits in the bovine sPs (114). In contrast, in another study immunoprecipitation with antibodies recognizing α4s revealed only constitutive subunits integrated into the murine complexes (115). Therefore, the composition of the mammalian spermatoproteasomes may differ and thus, should be additionally investigated. The proportion of proteasomes containing α4s compared to that of proteasomes containing constitutive α4 subunits in sperm could be as high as 80% (115). The presence of α4s in spermatoproteasomes, presumably, favors proteasome association with the activator PA200 (described below) (114). Spermatoproteasomes bearing PA200 activators were shown to participate in spermatogenesis and perform ubiquitin-independent degradation of acetylated histones (114).

Transcriptome analysis has indicated elevated expression of the *PSMA8* gene in large B-cell lymphoma, thymoma cells and testicular germ cell tumors (126). At the same time, the biological significance of α4s expression in cells outside of the testis remains to be elucidated. The presence of the α4s protein and spermatoproteasomes in the abovementioned tumors was not studied by itself, and the possibility of using α4s as a target for cancer therapy has also not been addressed.

## Proteasome Regulators

Almost two hundred 20S proteasome-interacting proteins have been discovered (8, 15). These can act alone or form large multisubunit complexes. Some are involved in the regulation of proteasome activity and substrate selection (8, 15, 47). The attachment of different regulators to 20S proteasomes comprises another level of cellular proteasome organization (3). Here, four major proteasome regulators, also known as activators and an endogenous proteasome inhibitor will be discussed. The roles of the 20S proteasome forms in cancer were described above irrespectively of any attached regulators; subsequently, the specific roles of proteasomes with regulators will be addressed.

### 19S (PA700)

Proteasomes can reversibly bind either one or two activators that “activate” them via opening of the 20S alpha gates (Figures 1, 2). Moreover, activators influence substrate selectivity of the proteasomes. Indeed, when 19S activator(s) (RP, PA700) are attached to the 20S proteasome, the proteasome-regulator complex (26S proteasome) is capable of specifically recognizing ubiquitinated proteins, cleaving the ubiquitin chains, unfolding and translocating substrates into the 20S core. At least 19 subunits are found in the 19S activator, which has dimensions of 150–160 to 180–200 Å and a molecular weight of ~700 kDa (127, 128). It is divided into the “base” and “lid” subcomplexes, assembled from subunits of two types: Rpt (Regulatory particle triple-A ATPase encoded by *PSMC1-6* genes) and Rpn (Regulatory particle non-ATPase encoded by *PSMD1-4,6-8,11-14* genes). The lid subcomplex performs the deubiquitination of substrates and contains the Rpn subunits 3,5,6,7,8,9,11,12, and 15, where Rpn11 is a Zn^2+^-dependent metalloisopeptidase that cleaves ubiquitin chains from tagged proteins (129, 130). The base subcomplex recognizes ubiquitinated proteins, performs substrate unfolding, binds to and facilitates the opening of the 20S proteasome gates. It is assembled from Rpn1, 2, 10, and the Rpt subunits Rpt1-6. The Rpn13 (ADRM1) is also frequently associated with the base subcomplex; however, it was not found incorporated into some analyzed 26S proteasomes (131, 132). Rpn1, 10 and 13 contact ubiquitin chains or proteins with ubiquitin-like domains (133–135). The Rpt subunits interact with the 20S proteasome and form a heterohexameric ring adjacent to its central pore. However, this interaction is not rigid, and current models indicate that the 19S activator is a dynamic structure (136, 137) and adopts different conformations following substrate engagement and ATP hydrolysis (128, 138–141). This generates pulling force, facilitates the unfolding and likely stepwise translocation of substrates into the 20S proteasome (138, 139). Efficient engagement and degradation require the presence of an unstructured region within the substrate, which seemingly interacts with the tyrosine pore loops of the Rpt subunits (142–144). The current understanding of substrate engagement and the translocation steps are described in Collins and Goldberg (144). Rpt subunits differ in their ability to bind and induce 20S proteasome gate opening (145–147). Critical roles are played by the Rpt subunits (Rpt2, 3, and 5), which contain evolutionarily conserved C-terminal HbYX motifs (145–147). These when inserted into the pockets between adjacent 20S alpha-subunits are believed to mediate rotation of the alpha-subunits, leading to gate opening and stabilization of the open-gate conformation (148). However, additional interactions with other activator subunits are also likely necessary to open the gates (132, 141, 149). Interestingly, certain proteins can be degraded by 26S proteasomes without ubiquitination (9, 150–154).

19S complexes associate with both constitutive and immune 20S proteasomes (Figure 1) (15). It was reported that 26S proteasomes with immune 20S cores play an essential role in the adaptation to the oxidative stress and demonstrate accelerated degradation of polyubiquitylated proteins compared to constitutive 26S proteasomes (85), however, these results have been challenged by another group (155). At the same time, additional confirmations that the iPs and the cPs have different substrate processing rates appeared later (80, 156, 157). Moreover, a remarkable mathematical model of the proteasome action was developed and allowed Liepe et al. to suggest that the transport of substrates into and out of the proteasome that varies between constitutive and immunoproteasomes is the rate-limiting step for the hydrolysis and explain the observed discrepancy (157). Interestingly, it was shown that the 26S immunoproteasome hydrolyzed unstructured polypeptides with 10-fold increased rates compared to 20S immunoproteasomes (158). Generally, the 19S activator binds 20S proteasomes more frequently than other activators. 26S proteasomes have been found in the cytoplasm and the nucleus and constitute 15–57% of all cellular proteasomes (5, 11, 13, 32).

Many cancer cells of different origins are highly dependent on and have elevated levels of 26S proteasomes, indicating that their survival relies on the maximal utilization of these proteasome forms (159). Indeed, the down-regulation of a single 19S subunit yielded significantly reduced viability in 14 of 19 tested cancer cell lines in comparison with normal cells (159). Moreover, aggressive and drug-resistant cancer cells were more affected by a reduction of 19S amounts, thus uncovering a novel opportunity for cancer therapy based on the inhibition of 19S subunits (159). The knockdown of Rpn2 induced cell cycle arrest in therapy-resistant breast cancer cells (160). However, glioma, breast cancer, and head and neck cancer cells with stem cell characteristics were reported to have decreased proteasome activity and reduced levels of the Rpn2 subunit of the 19S regulator but exhibited increased self-renewal capacity and tumorigenicity (161, 162). Interestingly, tumor cells with decreased proteasome activity were shown to be radioresistant, and the reduced expression of Rpn2 in tumors was associated with worse prognosis in radiotherapy-treated patients with head and neck cancer (162, 163). Moreover, naturally occurring decreased expression of any of 19S regulator subunits (either Rpn2, 7 or 11, PSMD5 or 10, or Rpt4) in many types of cancer has been associated with reduced sensitivity to proteasome inhibitors (164). Furthermore, in these cells, the downregulation of genes associated with EMT, EGF, and TNF signaling, and the upregulation of genes related to oxidative phosphorylation was observed (164). At the same time, cells with a reduction in 19S subunits and partial resistance to proteasome inhibitors were, surprisingly, found to be more sensitive to the BCL-2 family inhibitor ABT-263, highlighting the importance of combined therapeutic strategies for drug-resistant cancers (164).

### 11Sαβ (PA28αβ, REGαβ)

11Sαβ is the second most common cytoplasmic proteasome activator (5, 13, 165). Proteasomes containing 11Sαβ constitute between 1 and 40% of the cellular proteasome pool (Figure 1) (5). 11Sαβ is a 200 kDa protein complex that is 60 Å height and 90 Å in width with two coaxial openings (18 and 37 Å). It is assembled from four 11S alpha- and three 11S beta-subunits encoded by the *PSME1* and *PSME2* genes, respectively (166–168). Alpha and beta subunits both have MWs of ~28 kDa, share common structures and 47% sequence identity (169). Each subunit has four long alpha helices. The linker between helices 2 and 3 contains a so-called “activation loop” (170). 11S subunits have no HbYX motifs; however, their C-terminal sequences can insert into the pockets between the proteasome alpha-subunits and thus mediate binding to the 20S proteasome (171, 172). While binding to 20S CP is dependent on the C-termini of 11S subunits, proteasome activation is achieved through the induction of conformational changes in the proteasome alpha-subunits that are promoted by the activation loops (172). In contrast to the 19S complex, the 11Sαβ regulator was initially reported to be unable to facilitate the degradation of proteins but instead to stimulate the hydrolysis of peptides (173–176). Likewise to the 20S proteasome immune subunits, the synthesis of 11S α and β subunits is stimulated by IFN-γ (177–181). In addition, similarly to iPs, high levels of 11Sαβ were detected in various cells of the immune system (167, 182). Moreover, the interaction of immunoproteasomes and 11Sαβ seems to be common (15, 158). Hence, the role of 11Sαβ in antigen presentation, more specifically in the generation of MHC class I epitopes, has been proposed. However, till now the function/s of the activator are insufficiently understood (183). A lack of 11Sαβ synthesis in mice does not lead to severe abnormalities in immune responses against infections, but the expression of the activator was shown to stimulate the presentation of several MHC I epitopes (33, 165, 184). Nevertheless, the activator stimulated the production of epitopes for some MHC I alleles but downregulated the generation of ligands for others (185). Independent and additive effects of 11Sαβ and the immunoproteasome subunits on the production of MHC class I epitopes has been reported (186). In contrast, another study demonstrated that the 11Sαβ-20SiP complex, although produced several specific peptides, generally generated shorter peptide products and significantly fewer numbers of MHC I-compatible peptides than 20S or 26S iPs (158). Interestingly, it has been reported that 11Sαβ regulators favor interaction with iPs and intPs, indicating that the types of catalytic proteasome subunits influence regulator “preferences” (Figure 1) (15). However, there are data highlighting that 11Sαβ comparably associates with both constitutive and immune 20S proteasomes (33, 186).

In addition 11Sαβ seems to have functions besides antigen presentation. The decreased efficacy of oxidized protein degradation was detected in lysates from the activator knockout cells adapted to oxidative stress (41). Moreover, 11Sα subunit overexpression attenuates the increase in protein carbonyls and decreases the level of apoptosis in H_2_O_2_-treated cardiomyocytes (187). Finally, 11Sαβ directly stimulated the degradation of oxidized proteins by 20S proteasomes (188).

11Sαβ activator subunits are upregulated in prostate cancer (189), ovarian cancer (190), cervical cancer (191), renal cell carcinoma (192), oral squamous cell carcinoma (OSCC) (193). The overexpression of 11Sα was proposed as a prognostic factor in OSCC and was correlated with poorer prognosis and an increased risk of tumor recurrence (193). The siRNA-mediated inhibition of 11Sα led to reduced viability, colony formation, cell proliferation and invasion of OSCC cells *in vitro*, as well as reduced tumor size in BALB/C nude mice subcutaneously injected with modified cells (193). Concordantly, survival data from patients with renal cancer indicates that the high expression of 11Sαβ subunits is an unfavorable prognostic marker. In contrast, breast and thyroid cancer patients with increased expression of the activator subunits demonstrate longer survival [https://www.proteinatlas.org/ENSG00000092010-PSME1/pathology]. In addition, the downregulation of 11Sβ was reported in gastric cancer cells, and the decreased expression of 11Sβ was associated with the increased survival, proliferation and invasiveness of tumor cells (194, 195). Nude mice injected with gastric cancer cells with low expression of 11Sβ were shown to develop larger tumors than animals injected with 11Sβ-overexpressing cells (194). It was proposed that 11Sαβ may be involved in the degradation of the protein CLIC1, which can promote the migration and invasion of 11Sβ-deficient cancer cells via interactions with cytoskeletal proteins (194). The induction of 11Sαβ expression in cancer cells may either enhance or decrease the production and, consequently, the presentation of specific antigenic peptides. In melanoma 18a cells, the presentation of the tyrosinase-related protein 2 epitope TRP2_360−368_ was diminished in cells lacking 11Sαβ but was enhanced upon transfection of the cells with plasmids encoding subunits of the activator (196). Conversely, the upregulation of 11Sαβ decreased the generation of immunodominant MART-1_26−35_ epitopes in melanoma and HeLa cells (61). Thus, the role played by 11Sαβ in carcinogenesis seems to be different depending on the tumor type and likely the set of expressed cancer antigens.

### 11Sγ (PA28γ, REGγ)

Assembled from six equal subunits encoded by *PSME3* gene having MWs of 29.5 kDa, the 11Sγ activator is found mostly in the nucleus (197). The activation of the proteasome by 11Sγ resembles that by 11Sαβ and utilizes the activation loop. Interestingly, it mostly activates the trypsin-like activity of the proteasome (198–200). As reported in Fabre et al. (15) 11Sγ preferentially interacts with iPs, and ~5% of proteasomes from the cellular proteasome pool were shown to carry 11Sγ (5, 15). Using 11Sγ-deficient models, it was demonstrated that it is involved in the regulation of cellular growth and proliferation, cell cycle regulation, chromosomal stability during mitosis, G-protein-coupled receptor activity, energy and lipid metabolism, angiogenesis, the regulation of autophagy, immune reactions, angiogenesis, apoptosis, and cancer (197, 201–206). The pleiotropic effects of the activator may be explained by the 11Sγ-mediated, ubiquitin-independent degradation of important regulatory proteins (3), including steroid receptor coactivator-3 (SRC-3) (207), cyclin-dependent kinase inhibitors p21, p16, and p19 (208, 209), pituitary tumor transforming 1 (PTTG1) (210), HCV core protein (211), the insulin transcription activator MAFA (212), activation-induced deaminase-AID (202), casein kinase (CK) 1δ (204), the protein deacetylase SirT1 (203), protein kinase A (PKA) catalytic subunit-α (205), glycogen synthase kinase 3 beta (GSK3β) (213), the ribosomal DNA (rDNA) transcription activator SirT7 (214), the NF-κB inhibitor IκBε (206). Recent studies have demonstrated that 11Sγ mediates ubiquitin-independent degradation of the transcription factor c-Myc (215). However, in another study, it was shown that c-Myc degradation is enhanced in cells with silenced 11Sγ (216), indicating that the regulation of c-Myc stability by 11Sγ might involve several mechanisms. Interestingly, 11Sγ also stimulates MDM2-mediated p53 ubiquitination (217). Finally, similarly to 11Sαβ, 11Sγ was shown to facilitate the degradation of oxidized proteins (188).

The elevated expression of 11Sγ has been reported in several different cancers, including melanoma (218), squamous cell carcinoma (213), laryngeal carcinoma (219), lung, colorectal, thyroid, liver (220–222), endometrial (223), pancreatic (216), and breast cancer (224, 225), and is correlated with metastasis and poor prognosis in patients with liver, breast, and pancreatic cancer (216, 225–227). c-Myc is a transcription factor and a proto-oncogene that is overexpressed in many different tumors. The stability of c-Myc was increased by 11Sγ and was reported as a cause of stimulated glycolysis in pancreatic cancer cells (216). Another way that 11Sγ can affect c-Myc is through the regulation of β-catenin accumulation, which is an upstream regulator of c-Myc (213). 11Sγ expression is activated through the MAPK/p38/AP-1 signaling pathway and leads to the activation of Wnt/β-catenin signaling through enhanced degradation of GSK-3β, which is a negative regulator of β-catenin (213, 218). Interestingly, mutant but not wild-type p53 facilitated 11Sγ transcription and stimulated breast, colon and endometrial cancer cell proliferation (223, 228). At the same time, inhibition of the activator was shown to decrease the ubiquitination and degradation of wild-type p53 and thus promoted apoptosis in cancer cells (217). In addition, 11Sγ was shown to induce EMT in endometrial cancer cells (223). Therefore, since 11Sγ mediates the degradation of several regulatory proteins and thus plays an important role in carcinogenesis in many types of tumors, it represents a promising therapeutic target. Indeed, the downregulation of 11Sγ inhibited cell proliferation, induced apoptosis in different cancer cell types and attenuated the growth of melanoma, adenocarcinoma, and pancreatic cancer in nude mice (213, 216–218, 223). Interestingly, 11Sγ mRNA translation was found to be regulated by endogenous microRNAs, such as mir-7 in breast and non-small-cell lung cancer (229, 230) and miR-195-5p, which was shown to suppress the β-catenin pathway and to reduce the proliferation of renal cell carcinoma cells and increase their sensitivity to a kinase inhibitor drug (231). Finally, the anti-tumor activity of energy metabolism inhibitors was increased in mice injected with colorectal carcinoma cells with 11Sγ knockdown (214).

### PA200

The PA200 is a large 200 kDa phosphoprotein a product of the *PSME4* gene expression and a second nuclear-specific proteasome activator. It has a solenoid conformation and visually resembles a hat or a slanting dome, and it is ~60 Å in height and 100 Å in width (232–235). PA200 or Blm10 (yeast homolog) binding induces structural changes that produce partially open conformations in 20S CP gates (234, 236). Three C-terminal residues in Blm10 (TyrTyrAla) match the HbYX formula and bind in the lysine pocket between the α5 and α6 subunits of the 20S complex. The penultimate Tyr2142 forms a hydrogen bond with α5Gly19 and stabilizes the adjacent α5Pro17 reverse turn in the open gate conformation (236). However, binding of a single HbYX motif may be insufficient and secondary interactions allow Blm10 to partially open the gate (237). Label-free quantitative mass spectrometry analysis of 9 different cell lines revealed that <5% of 20S proteasomes bear PA200 regulators (5). Another study demonstrated that ~8% of proteasomes in muscle cells and up to 89% in the testis are attached to PA200 (Figures 1, 2) (114). Interestingly, recruitment of PA28γ and PA200 to 20S and 26S proteasomes following proteasome inhibition was recently reported (238), indicating that the number of proteasomes with PA200 is dependent on cell condition and proteasome activity. Constitutive 20S proteasomes are likely to be more frequently associated with PA200 than immunoproteasomes (15). Interestingly, PA200 stimulates proteasome caspase-like activity more than other types of activity (232). PA200 is required for DNA repair and normal spermatogenesis in testes via promotion of the ATP- and ubiquitin-independent degradation of acetylated histones (114, 232, 239, 240).

The high expression of PA200 is an unfavorable prognostic marker in liver and endometrial cancer [https://www.proteinatlas.org/ENSG00000068878-PSME4/pathology]. The role of PA200 in cancer is mostly associated with DNA repair mechanisms, specifically the degradation of acetylated histones (114, 240). Indeed, cervical cancer cells lacking PA200 demonstrated increased sensitivity to ionizing radiation (241). Interestingly, proteasomes with PA200 are involved in the maintenance of glutamine homeostasis and, in conditions of increased glutamine demand in cells after radiation exposure, supply additional glutamine through the elevation of caspase-like activity and thus affect the long-term survival of tumor cells (241, 242). Moreover, PA200-depleted cells are unresponsive to decreased levels of glutamine and continue to grow in conditions in which cells with normal levels of the activator stop proliferating (242).

### PI31

Comparing with 19S, 11S, and PA200 activators PI31 is the least frequent regulator associated with proteasomes and the amounts of 20S bound to PI31 are substoichiometric according to Fabre et al. (5). Hence it is not surprising that among mentioned proteasome regulators the PI31 is the most poorly studied. Despite that it was discovered back in 1992 by DeMartino group (243), till now there are several unresolved questions regarding its cellular function and properties. The PI31 was characterized due to its ability to decrease 20S proteasome activity *in vitro* (243) and compete for the 20S binding with 19S and 11S regulators (244). However, it was demonstrated that in transfected cells PI31 does not inhibit cellular proteasome activity (245). Encoded by the *PSMF1* gene PI31 represents a 31 kDa protein composed of N-terminal globular domain and C-terminal domain (244). The C-terminal domain of PI31 is rich in prolines, has intrinsically disordered structure and bares a proteasome-activation HbYX motif, characteristic for 19S and PA200 proteasome regulators (246, 247). The PI31 can act as a monomer, but also form homodimers (243, 244), possibly larger multimers (243, 248) and even heterodimers with other proteins (249). Which PI31 complexes are more prone to interact with 20S proteasomes is still unclear. The PI31 was shown to directly interact with triple-A ATPase valosin-containing protein (VCP) which may counteract the 20S proteasome inhibition by PI31 (248). Another interesting insight into the function of PI31 came from experiments with *Drosophila* homolog DmPI31. Bader et al. demonstrated that DmPI31 is necessary for a proper proteasomal function, sperm differentiation and forms complexes with the F-box protein Nutcracker, a component of E3 ubiquitin ligase SCF complex (246). Surprisingly, PI31 was stabilized by Nutcracker. Moreover, ectopic expression of PI31 has been shown to rescue *Drosophila* phenotypes caused by impaired proteasome function and increase the activity of bovine 26S proteasomes *in vitro*, indicating its role in proteasome activation (246). These investigations were continued by Cho-Park and Steller who has shown that DmPI31 interacts with ADP-ribosyltransferase tankyrase (TNKS) (250). TNKS was demonstrated to perform ADP-ribosylation of the DmPI31, reducing its affinity to alpha subunits of the 20S proteasome, therefore blocking the inhibition of the complex and modulating proteasome activity. In addition, authors demonstrated that ADP-ribosylated DmPI31 stimulated 26S proteasome activity by promoting its assembly from 20S cores and 19S particles. Comparing to non-modified ADP-ribosylated DmPI31 had increased ability to interact with 19S assembly chaperones, liberating the regulatory particle for the interaction with the 20S proteasomes (250). However, these results were contradicted by more recent studies (247) showing no effect of PI31 on the intact 26S proteasome activity in *in vitro*. Moreover, in addition to blocking 20S-19S binding, PI31 has been shown to inhibit the assembly of 19S from subcomplexes *in vitro*, in cells, however, overexpression, or reduced expression of PI31 did not affect proteasome activity, indicating that PI31 demonstrates different effects *in vitro* and *in cellulo*. Finally no effect of the ribosylation inhibitor on 26S content and function was observed (247). Interestingly, PI31 was demonstrated to negatively affect maturation of immunoproteasomes and MHC class I presentation of an immunoproteasome-dependent epitope (245). Finally, it was shown that the majority of cellular PI31 were not associated with proteasomes (247). On the other hand, if it is, however, attached to the proteasome, according to Fabre et al., it prefers constitutive 20S complexes (15). In general, the precise mechanisms of proteasome regulation by PI31 should be further investigated and prudently addressed.

The role of PI31 in cancer is insufficiently studied. Though it is expressed in prostate, ovarian, colorectal, endometrial, renal, breast, liver cancers and malignant gliomas, its prognostic value is limited [https://www.proteinatlas.org/ENSG00000125818-PSMF1/pathology]. At the same time, methylation-dependent downregulation of PI31 expression has been shown in breast cancer cells (251). Moreover, PI31 was demonstrated to negatively influence the iPs maturation and presentation of certain epitopes as well as to reduce the numbers of MHC class I molecules on the cellular surface following IFN-γ stimulation (245). Thus, it might be expected that PI31 expression may affect the recognition of tumors by the cytotoxic lymphocytes. Another peculiarity of PI31 which may be relevant to cancer is its association with the VCP (248).

### VCP

VCP/p97 is a member of AAA-ATPase family, it contains two nucleotide binding domains and assembles in a double-ring hexamer with MW around 600 kDa (252, 253). VCP constitute up to a 1% of cytoplasmic protein mass (254) it is also found in nucleus (255). VCP together with ~30 cofactors is playing important roles in various basic metabolic processes including: ER-associated degradation, unfolded protein response, chromatin-associated degradation, ribosomal-associated degradation, mitochondria-associated degradation, autophagy, aggregate dissociation, lipid droplet biogenesis, endosomal trafficking, mitochondrial fusion as well as ER, and Golgi assembly (256–259). These properties are based on the ability of VCP-cofactor complexes to recognize and unfold ubiquitinated substrates via translocation through a central pore of the VCP hexamer (260, 261). Thus, VCP induces separation of individual polyubiquitinated substrates from membranes or binding partners, often followed by degradation of these substrates by the 26S proteasome (260). However, recent findings demonstrate that VCP and its yeast homolog Cdc48 can associate directly with 20S proteasome in a manner similar to the way that the activators do. Indeed C-terminal tail of Cdc48 contains the HbYX motif. In archaea 20S cores associate with PAN or Cdc48 (262). Barthelme et al. demonstrated that mouse VCP interacts with mouse 20S core and enhances fluorogenic peptide cleavage by the complex. Interestingly using archaeon 20S and Cdc48 authors demonstrated that interaction between the complexes is bipartite and involves interactions of loops near the bottom of Cdc48 axial channel with N-terminal residues of 20S proteasome alpha subunits, as well as HbYX-dependent interactions (263). The stoichiometry of VCP-20S complexes in mammalian cells was not accurately determined, although if, indeed, these complexes are formed, their association with 20S complexes may be transient and requires artificial stabilization in order to be detected (264, 265). In contrast to 19S, 11S, or PA200 activators, the effects mediated by VCP seems more frequently associated with the complex itself rather than with the VCP-20S proteasome.

The pleiotropic functions and involvement in the maintenance of protein homeostasis attracted much attention to the VCP as a putative anticancer therapy target (266, 267). Indeed, the VCP is upregulated in many different tumors (colorectal, gastric, hepatocellular, breast, non-smal-cell lung, and esophagal squamous cell carcinomas, pancreatic endocrine cancer, prostate and follicular thyroid cancers) and its expression is associated with the poor prognosis (268, 269). Interestingly, the molecular mechanisms by which VCP promotes cancer growth, progression and invasion are at least in part associated with stimulation of UPS-mediated degradation of important regulatory proteins including IκB (NFκB inhibitor) (270–272) and p53 (272, 273). Recently, an important role of VCP in maintaining cancer cell homeostasis in conditions of nutrient (glutamine) depletion was reported (274). Inhibition of VCP induces cancer cell growth arrest and apoptosis (272, 273). In this regard, several VCP inhibitors were developed (266, 275). Recently Andersen et al. reported that a small molecule CB-5083 is an effective inhibitor of VCP witch induces proteotoxic stress, activates unfolded protein response leading to the apoptosis of affected cells (276). CB-5083 demonstrated high antitumor activity which was shown both using cancer cell lines (350) and in xenograft tumor models. Interestingly, CB-5083 was more effective against solid tumors than proteasome inhibitor bortezomib (276). CB-5083 could be delivered orally and was the first VCP inhibitor tested in clinical trials. However, the trials were terminated due to the “unexpected off-target effect” indicating that more specific VCP inhibitors are necessary (277).

### Hybrid Proteasomes

**Figure 3:**
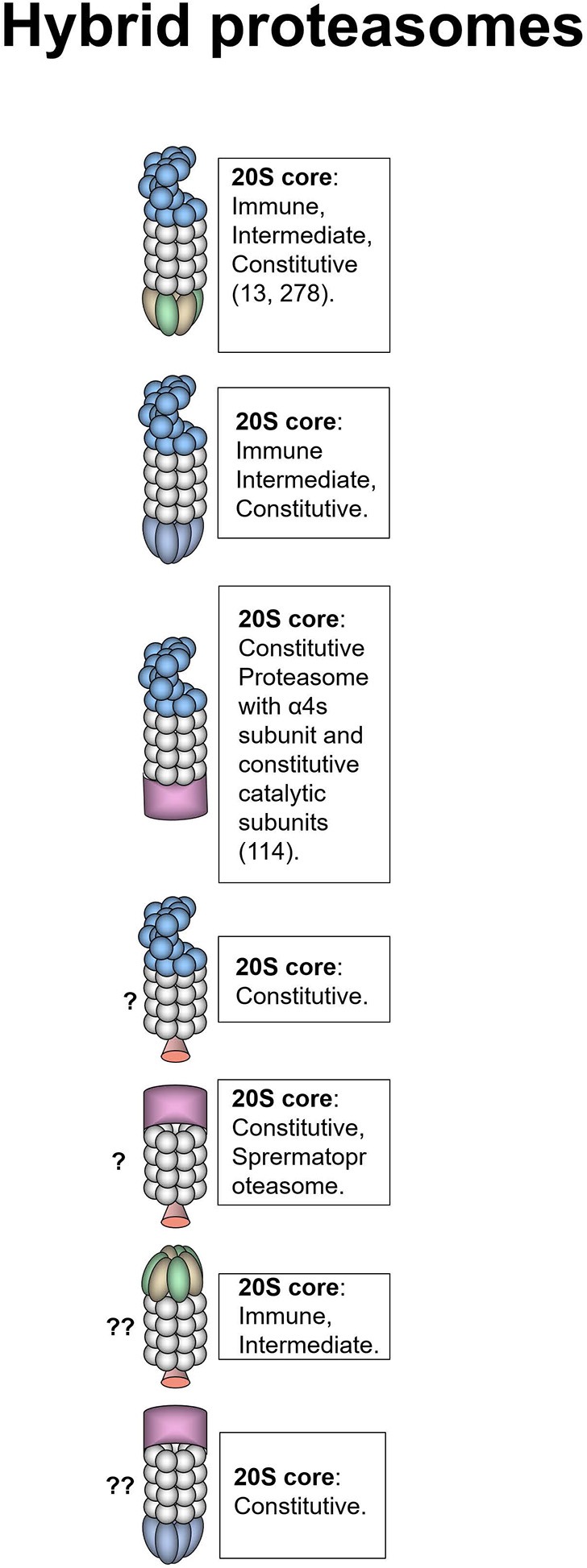
Hybrid proteasomes. These proteasomes represent complexes of two different activators attached to a single 20S particle. Initially hybrids with 19S-20S-11Sαβ and 19S-20S-11Sγ architecture were identified (278). In certain cells 19S-20S-11Sαβ can represent up to 24% of all proteasomes (13). 19S-20S-11Sγ complexes are less frequent (278). 19S-20S-11Sαβ proteasomes are localized in the cytoplasm whereas 19S-20S-11Sγ–in the nucleus. The 20S core particle in 19S-20S-11Sαβ complex is likely immune or intermediate, this is confirmed by the increase of such complexes following IFN-γ treatment (13, 278), still cases with constitutive 20S could not be ruled out (13). 19S-20S-11Sγ could also contain constitutive 20S particle. Another form of hybrid proteasome is the 19S-20S-PA200 (232). The 19S-20S-PA200 proteasomes have nuclear localization, the 20S core particle in these hybrid complexes could be constitutive, or contain α4s subunits (114). Theoretically other hybrid proteasomes: 19S-20S-PI31, PA200-20S-PI31, PA200-20S-11Sγ, and 11Sαβ-20S-PI31 can also exist, although were not revealed so far. Question mark indicates that the presence of a particular proteasome form is uncertain.

The symmetry of the 20S proteasome allows binding of two activators to the same core particle and frequently these activators are different. Such proteasomes are called hybrid proteasomes. Hybrid proteasomes as it is and of 19S-20S-11Sαβ and 19S-20S-11Sγ composition were first described by Hendil et al. in 1998 (Figure 3) (278). Tanahashi et al. quantified that up to 24% of proteasomes in Hela cells are 19S-20S-11Sαβ (13). The function of this type of hybrids is likely ubiquitin-dependent degradation of protein substrates (13). However, hybrid proteasomes produce different pattern of peptides comparing to classical 26S proteasomes (279, 280). According to the intracellular localization of the activators these hybrids are localized in the cytoplasm (19S-20S-11Sαβ) and in the nucleus (19S-20S-11Sγ). The 20S core particle in 19S-20S-11Sαβ complex is likely immune or intermediate. This was confirmed by the increase of such complexes following IFN-γ treatment (13, 278), still cases with constitutive 20S could not be ruled out (13). We also can expect that these hybrid proteasomes may be generated following the oxidative stress (41). 19S-20S-11Sγ complexes are less frequent (278) and likely contain constitutive 20S cores. The 19S-20S-PA200 hybrid proteasomes were first reported by Ustrell et al. (232). Subsequently hybrid proteasomes with 19S-20S-PA200 structure were discovered in yeasts where Blm10 was found associated with 19S-20S proteasome (281). Blickwedehl and coauthors demonstrated increased amount of 19S-20S-PA200 hybrid proteasomes in HeLa cells following treatment with ionizing radiation (241) indicating a role of these proteasome in DNA damage response. As discussed above, PA200 bearing proteasomes are involved in ubiquitin-independent acetylation-associated degradation of core histones in spermatogenesis and response to DNA damage (114). 19S-20S-PA200 proteasomes have nuclear localization, the 20S core particles in these complexes likely contain constitutive catalytic subunits, additionally α4s subunit can be present in certain germ cells (114). It worth mentioning that 11Sαβ-20S-PA200 hybrid proteasome was recently reported by Erokhov et al. in the rat brains (16). The existence of such hybrid proteasome is unexpected since 11Sαβ and PA200 normally have different subcellular localization. The existence of hybrid proteasomes of 19S-20S-PI31, PA200-20S-PI31, PA200-20S-11Sγ, 11Sαβ-20S-PI31, or complexes with VCP was not demonstrated so far, still could not be excluded.

Generally, the specific function of hybrid proteasomes in cancer is unknown.

## Concluding Remarks

**Figure 4:**
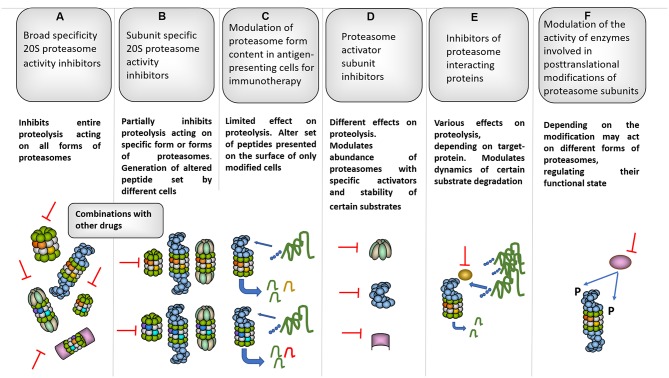
Several proteasome-based strategies for cancer therapy. **(A)** Broad specificity proteasome inhibitors for instance bortezomib affect all forms of proteasomes, thus influencing entire proteolysis in different cells (98). Along these lines, bortezomib is known for strong side-effects that limit its clinical use. To decrease the side-effects and increase the efficacy combinations of bortezomib with other molecules were proposed (283, 284). **(B)** Subunit specific inhibitors allowed targeting specific subsets of proteasomes and especially iP subunit-specific inhibitors are considered very useful against certain autoimmune disorders and inflammation-induced tumors (90, 91). Moreover, generally they should be safer since have limited effect on overall proteolysis in various cells where cPs dominate the proteasome pool. At the same time, constitutive and iP subunit inhibitors were shown to induce EMT, thus special care should be taken when the therapy is concerned. Interestingly, certain inhibitors were shown to increase cancer cell sensitivity to iP subunit–specific inhibitors (102). Importantly, such inhibitors may differently affect generation of tumor antigenic peptides influencing (in both directions) immune recognition of affected cells. **(C)** This may be further utilized in a method based on *ex vivo* approach with modification of proteasome subunit expression in antigen-presenting cells either using siRNA or CRISPR/Cas technology. Immunotherapy using this kind of cells transfected with cancer antigens allowed efficient generation and presentation of particular antigenic peptides which are better generated by a particular proteasome form as well as further reduction of side effects (62). **(D)** Inhibition of activators represent an additional strategy and may be used to target aggressive tumor cells with high 19S expression as well as to disrupt glucose metabolism affecting 11Sγ (216) and increase radiosensitivity in case of PA200 inhibition (242). **(E)** Furthermore, several proteasome-associated proteins with proteasome-regulatory functions may serve as targets for cancer therapy. For example: deubiquitinase Usp14. Inhibition of Usp14 can lead to prolonged association and, thus better degradation of certain substrates by the proteasome (285) and cause ubiquitin deficiency (286). Moreover, Usp14 regulates 26S proteasome function and its association with proteasomes is stimulated by ubiquitinated proteins (287). This logically is important for cancer cells and, concordantly, the inhibition of Usp14 lead to decreased growth of different tumors (288, 289). **(F)** Finally, the proteasome diversity is expanded by different post-translational modifications which can regulate proteasome function, activity and processivity (7, 290), thus, blocking of the responsible enzymes represents additional promising strategy to fight different cancers (291).

Increased metabolic activity and the need to adapt to various stresses explain why many proteasome genes are upregulated in different tumor types. Thus, cancer cells are frequently more dependent on appropriate UPS function than normal cells, making the system an appealing target for cancer therapy. Accordingly, proteasome form diversity allows the development of new and the fine-tuning of known approaches for cancer management. For instance, the quantity and ratio of proteasome forms (constitutive proteasomes vs. immunoproteasomes) in tumor cells can predict the clinical effects of broad specificity proteasome inhibitors (99, 282), which target both the constitutive and immune catalytic subunits of 20S proteasomes and represent major UPS-directed anti-cancer drugs that are used in clinics (17, 98). However, the efficacy of such inhibitors is limited by side effects and resistance; therefore, the continuous development of novel inhibitors, including subunit- and thus, form-specific ones is currently a hot topic in molecular medicine (Figure 4) (18, 98, 292). At the same time, care should be taken when proteasome inhibitors are used in therapy, since they can induce EMT and promote tumor growth and expansion (57, 101). In addition, cancer stem cells are characterized by decreased proteasome activity and increased resistance to proteasome inhibitors (293), indicating that the improvement of inhibitor-based therapeutic approaches is necessary. Indeed, combinations of inhibitors with different molecules decreased the active dose required and increased inhibitor potency (283, 284). Different proteasome forms found in cancer cells are involved in the immune recognition of the tumor. Through the production of antigenic peptides, different proteasomes may either stimulate or decrease the recognition of tumor cells by T cells. This creates another motivation for the utilization of form-specific inhibitors. In addition, the manipulation with proteasome forms in antigen-presenting cells was effectively used in cancer immunotherapy (Figure 4) (62). Moreover, proteasome activators are promising targets for the therapy of several tumors. The use of microRNA-based strategies is a new concept that is being explored for cancer treatment (294) and activator targeting by endogenous miRNAs might be an appropriate approach (Figure 4). Furthermore, the diversity of proteasomes can be increased by hundreds of proteasome-interacting proteins including the Ecm29 protein, the deubiquitinating enzymes Usp14, and Uch37, which have all either studied as or could become novel therapeutic targets (Figure 4) (267, 288, 295–298). For example, Usp14 overexpression was observed in lung, breast, pancreatic, gastric, and endometrial cancer (289, 295, 299) and its inhibition via RNA interference or small molecule inhibitors resulted in reduced proliferation invasion and increased apoptosis in lung, breast, pancreatic, prostate, endometrial cancer, and melanoma cells (288, 289, 295, 296). The downregulation of Usp14 ensured smaller tumor sizes and longer survival in nude mice injected with lung cancer or melanoma cells (288, 289). Finally, proteasome heterogeneity is increased by the post-translational modifications of proteasome subunits, which have a significant impact on the functional activity of different forms of proteasomes (7, 290), therefore; the enzymes that are involved also represent attractive targets for cancer treatment (291).

Overall, the diversity of proteasome forms and the complexity of UPS provide exciting opportunities to optimize and fine-tune cancer therapy.

## Author Contributions

AM: writing—original draft preparation. AM and VK: writing—review and editing and funding acquisition.

### Conflict of Interest Statement

The authors declare that the research was conducted in the absence of any commercial or financial relationships that could be construed as a potential conflict of interest.
